# Acute Necrotizing Encephalopathy Associated With SARS-CoV-2 Exposure in a Pediatric Patient

**DOI:** 10.7759/cureus.15018

**Published:** 2021-05-13

**Authors:** Claudia Lazarte-Rantes, Julissa Guevara-Castañón, Lelia Romero, Daniel Guillén-Pinto

**Affiliations:** 1 Pediatric Radiology, Instituto Nacional de Salud del Niño San Borja, Lima, PER; 2 Pediatric Radiology, Resocentro, Lima, PER; 3 Pediatric Neurology, Hospital III "José Cayetano Heredia", Piura, PER; 4 Radiology, Resocentro, Lima, PER; 5 Pediatric Neurology, Hospital Cayetano Heredia, Universidad Peruana Cayetano Heredia, Lima, PER

**Keywords:** acute necrotizing encephalitis, child, covid-19, sars cov-2

## Abstract

We present the case of a nine-month-old male child with three days of fever, irritability, left focal seizure, and febrile focal status epilepticus. He had no history of previous comorbidities. A lumbar puncture was performed, which showed cerebrospinal fluid (CSF) leukocytosis; protein and glucose were normal, and the polymerase chain reaction (PCR) panel for 14 pathogens in CSF was negative. Immunoglobulin G (IgG) qualitative and quantitative tests were positive for coronavirus disease 2019 (COVID-19) upon arrival. An MRI performed one week after the initial onset showed findings suggestive of acute necrotizing encephalopathy (ANE). The patient required mechanical ventilation. However, his symptoms did not improve and follow-up imaging two weeks later showed progression of the disease with hemorrhagic changes. To our knowledge, this is the first reported case of ANE associated with severe acute respiratory syndrome coronavirus 2 (SARS-CoV-​2) infection in a pediatric patient.

## Introduction

The most common viral pathogens associated with respiratory infections in humans are influenza, human metapneumovirus, respiratory syncytial virus, and coronavirus [1]. The invasion of viral respiratory pathogens into the central nervous system (CNS) requires some degree of neurotropism [2]. For acute neurologic diseases, the neuropathogenesis of coronavirus disease 2019 (COVID-19) has been described as associated with cases of encephalitis, Guillain-Barré syndrome (GBS), acute disseminated encephalomyelitis (ADEM), acute flaccid myelitis, acute cerebrovascular disease, and necrotizing encephalopathy during infection, as well as symptomatic manifestation with headache, anosmia/hyposmia, ageusia, vision loss, neuralgic pain, myalgia, insomnia, dizziness, and status epilepticus [3,4].

It is believed that severe acute respiratory syndrome coronavirus 2 (SARS-CoV-​2) enters into human cells by binding to angiotensin-converting enzyme receptors, and hence the cells expressing this receptor are the most vulnerable to injury [5]. Among them, glial cells and neurons, as well as endothelial and arterial smooth muscle cells in the brain, are prone to neurotropic damage. Mechanisms of entry can be through hematogenous dissemination via endothelial cells or via the cribriform plate and olfactory bulb [6]. However, CNS involvement can also be due to a “cytokine storm” similar to hemophagocytic lymphohistiocytosis with consequent sepsis and multiorgan failure [7].

COVID-19 infection is known to principally affect the respiratory system, with adults being more vulnerable than children, and its neurotropic characteristics have been frequently reported. However, in our case, the ANE was thought to be an autoimmune mechanism as immunoglobulin G (IgG) was positive and polymerase chain reaction (PCR) for COVID-19 was negative, which was in line with other cases reported in patients with encephalitis [8].

## Case presentation

We present the case of a nine-month-old male child, who had been born by cesarean section due to macrosomia with a head circumference at p50, with normal motor development. He was brought to the emergency room with three days of fever of 38 °C and irritability. On the third day of the disease onset, he presented a left clonic focal seizure lasting 30 seconds with a rectal temperature of 38.5 °C. Seven hours later, he presented two left clonic focal seizures lasting one minute. He was admitted to the emergency room with a rectal temperature of 39.5 °C, head circumference at p50, no skin lesions, and episodes of irritability and drowsiness, global hypotonia, hyperreflexia, and no meningeal signs; photomotor reflex was present and no cranial nerves abnormalities were observed. The laboratory results showed leukocytosis with neutrophilia and positive qualitative and quantitative IgG for COVID-19.

On the fourth day of the disease onset, he presented stupor, fever of 40 °C, left focal febrile seizure lasting more than five minutes, leading to febrile focal status epilepticus. The management of epileptic state was started, and it was controlled with 7 mcg/kg/minute of midazolam infusion, intubation, and ventilatory support. Cerebrospinal fluid (CSF) analysis showed mild leukocytosis; protein and glucose were normal and the PCR panel for 14 pathogens in CSF were negative (Table 1).

**Table 1:** CSF analysis of the patient CSF: cerebrospinal fluid

Variables	Results
Color	Cristal rock
Leukocytes	8 cells/mm^3^
Protein	27 mg/dl
Glucose	82 mg/dl
Red blood cells	0
Gram stain	Negative
Chinese ink	Negative
Ziehl–Neelsen staining	Negative
Culture	Negative
Escherichia coli	Not detected
Haemophilus influenzae type B	Not detected
Listeria monocytogenes	Not detected
Neisseria meningitidis	Not detected
Streptococcus agalactiae	Not detected
Streptococcus pneumoniae	Not detected
Cytomegalovirus (CMV)	Not detected
Enterovirus	Not detected
Herpes simplex virus 1	Not detected
Herpes simplex virus 2	Not detected
Human herpesvirus 6	Not detected
Parechovirus	Not detected
Varicella-zoster virus	Not detected
Cryptococcus neoformans/gattii	Not detected

On the sixth day of the disease onset, his seizures were in control and mechanical ventilation was stopped. At the follow-up, he presented clinical signs of autonomic dysfunction. He persisted with the compromise of the sensorium, necessitating the initiation of treatment with immunoglobulins 4 gr/day for five days. At 72 hours after the beginning of treatment, a slight improvement was observed in the neurological exam, and the patient became hemodynamically stable.

As in other tests carried out in patients with COVID-19, our patient had an increase in D-dimer and lactic dehydrogenase but with a normal value of ferritin and normal dosage of vitamin D. Dosage quantitative IgG COVID-19 showed a cut-​off index (COI) of 62.5 [method: time-resolved immunofluorometric assay (TR-IFMA)]. RT-PCR for SARS-CoV-2 detection in nasopharyngeal swab was negative. PCR for influenza detection was also negative (Table 2).

**Table 2:** Laboratory test results of the patient COVID-19: coronavirus disease 2019; IgM: immunoglobulin M; IgG: immunoglobulin G; SARS: severe acute respiratory syndrome; RT-PCR: reverse transcription-polymerase chain reaction; COI: cut-off index

Test	Value	Units	Referential values
Total and fractionated protein	8.56	g/dL	6.1-7.9
Albumin	5.1	g/dL	3.5-4.8
Globulin	3.39	g/dL	2.6-3.1
Lactic dehydrogenase	307	U/L	135-225
D-dimer	1.57	µg/ml	0.06-0.70
Ferritin	139.50	ng/ml	20-200
Vitamin D (25-hydroxyvitamin D)	115.60	ng/ml	20-160
T.qualitative COVID-19	IgG (+)		
Dosage quantitative IgM COVID-19	0	COI	≥1
Dosage quantitative IgG COVID-19	62.5	COI	≥1
SARS-coronavirus RT-PCR (respiratory)	Negative		
PCR influenza	Negative		

**Figure 1:**
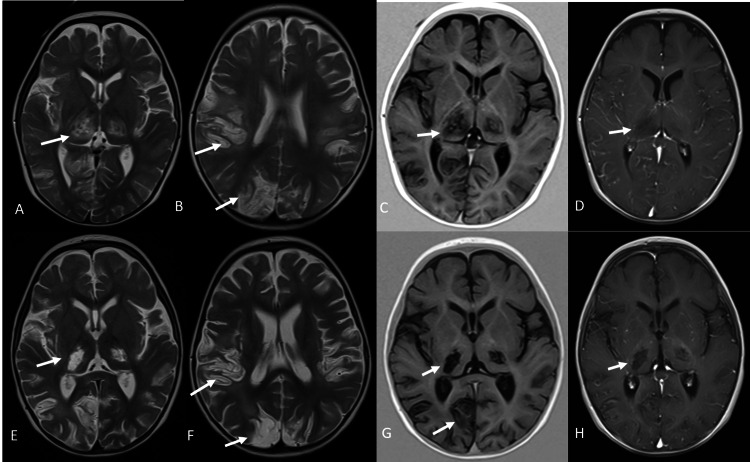
First and second brain MRI Axial T2-weighted images (A and B) and the axial T1-inversion recovery image (C) show necrosis of both the thalamus and at the cortico-subcortical junction of both parietal and occipital lobes with a lack of contrast enhancement (D). Images E-H show the evolution of the previous findings two weeks later with the loss of parenchyma MRI: magnetic resonance imaging

**Figure 2:**
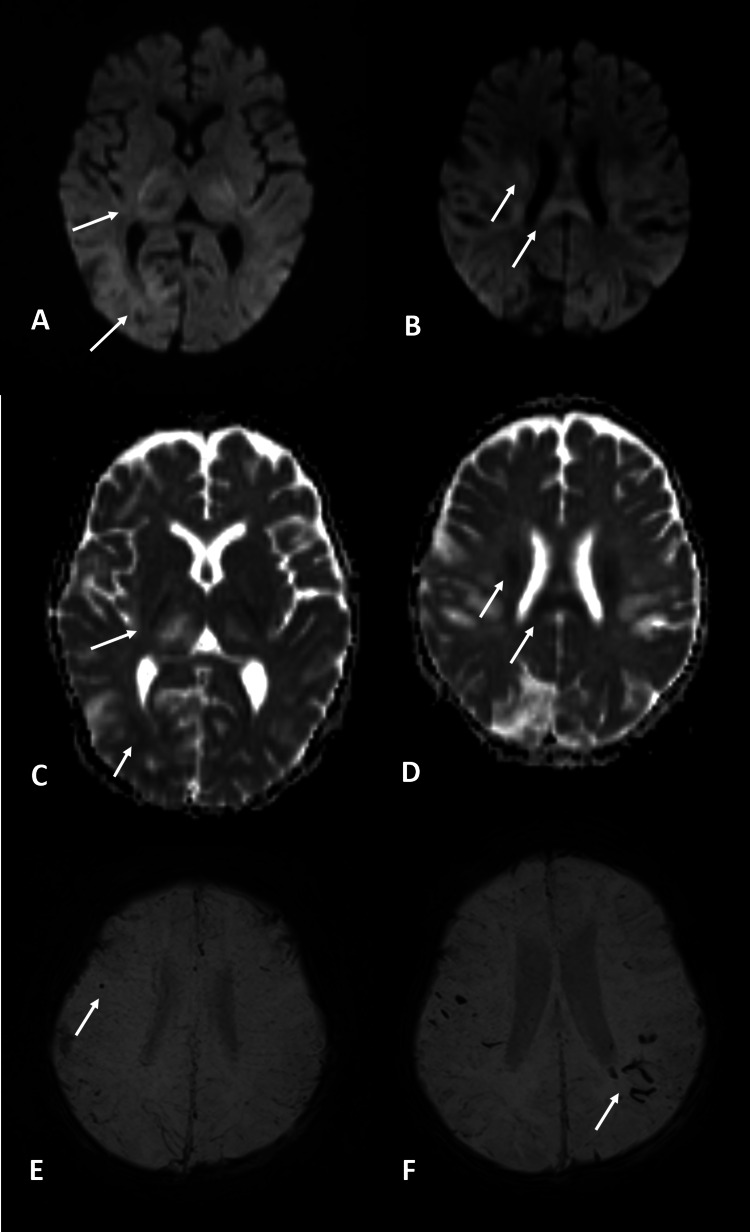
MRI – diffusion and SWI Diffusion (A, B) and ADC map (C, D) at the initial exam show restriction in the posterior limb of the internal capsule, cortico-subcortical junction of the occipital lobes (A and C), the posterior body of the corpus callosum, and corona radiate (B and D). A little dot of hemorrhage was visualized in the frontal lobe at the initial exam as a blooming artifact in the susceptibility-weighted image (E). In the follow-up exam, more hemorrhagic changes were visualized in the parietal lobes (F) MRI: magnetic resonance imaging; SWI: susceptibility-weighted imaging; ADC: apparent diffusion coefficient

A brain MRI was performed on the 15th day of the disease onset, which showed areas of necrosis at the thalami and at the cortico-subcortical junction in the parietal and occipital lobes with no contrast enhancement (Figure 1). Areas of restricted diffusion were seen on the posterior limb of the internal capsule, the posterior body of the corpus callosum and corona radiata, as well as at the cortico-subcortical junction of occipital lobes (Figure 2). A follow-up brain MRI two weeks later showed loss of parenchyma on previously affected areas as well as a blooming artifact on susceptibility-weighted images, suggestive of hemorrhagic changes that were very discreet on initial images (Figures 1, 2).

The patient was discharged awake with few episodes of irritability; he had quadriparesis, axial hypotonia, appendicular hypertonia, and global hyperreflexia. He had not achieved head and trunk motor control by the time of discharge.

## Discussion

ANE associated with COVID-19 has been reported in very few cases among the adult population [4,9,10]. Although some pediatric cases have been reported with acute encephalopathy, edema, and acute ischemic stroke associated with coronavirus disease [11-14], to our knowledge, this is the first reported case of a child with positive IgG of SARS-CoV-2 and ANE. 

Although most recent theories on the physiopathology of ANE suggest an immune-mediated or metabolic cause, it has been frequently associated with viral agents such as influenza A, mycoplasma, or herpes simplex virus [13,15]. The clinical onset is rapid and progressive with a poor prognosis, and neurological symptoms are non-specific, but most frequently involve seizures and impaired consciousness as in our case.

MR features consist of multiple T2-hyperintense symmetrical lesions most frequently found in the thalami as well as putamina, periventricular white matter, cerebellum, and brain stem tegmentum. Hemorrhage and cavitation may also be present. The extent of lesions on MRI has been found to correlate to a worse prognosis, particularly when the brain stem is involved, as well as the presence of hemorrhage or tissue loss, such as in our case. Most frequent MR findings on pediatric patients with neurological manifestations of COVID-19 include signal changes in the splenium consisting of T2-hyperintense lesions with or without restricted diffusion, most likely secondary to multisystem inflammatory syndrome [8]. Involvement of the genu and centrum semiovale has also been described. Among the differential diagnosis of multiple symmetric T2-hyperintense lesions with thalamic involvement are toxic encephalopathy, hemolytic uremic syndrome, and hemorrhagic shock [15,16].

We observed elevated levels of lactate dehydrogenase and D-dimer; previous studies involving infections caused by COVID-19 have routinely reported the elevation of these clinical markers [17]. Zhou et al. reported that an elevated D-dimer (>1 ug/L) at admission is a risk factor for death in adult patients with COVID-19 [18]. Typical abnormal laboratory findings were also reported, such as elevated D-dimer, in a cohort of pediatric patients with COVID-19. Also, an elevated D-dimer level was more frequent in infants (zero to one year in age) than in the other age groups, and this may suggest that infants might be more seriously affected by COVID-19 than older children [19].

The limitation of this report is that we could not obtain positive PCR for SARS-CoV-2 at the initial stage of the disease in the patient. However, due to the clinical manifestations and MRI findings, we postulate that this case represents an association between ANE and SARS-CoV-2 exposure due to autoimmune-mediated mechanisms similar to other case reports in adults. Likewise, there was an epidemiological antecedent of two relatives who shared the same physical environment with the infant, and they were also found to be positive for IgM and IgG in the qualitative test for COVID-19.

## Conclusions

ANE associated with SARS-CoV-2 due to autoimmune-mediated mechanisms has been described in the adult population. Children could also be at risk of developing this disease following COVID-19 infection. Pediatricians need to be aware of this association in pediatric patients with COVID-19 presenting to the emergency room with neurologic symptoms and clinical worsening. This new case adds to the new body of evidence related to CNS involvement in SARS-CoV-2, and further studies are required to gain more insight into this.
